# Identification of Hub Genes Associated With Immune Infiltration and Predict Prognosis in Hepatocellular Carcinoma via Bioinformatics Approaches

**DOI:** 10.3389/fgene.2020.575762

**Published:** 2021-01-11

**Authors:** Huaping Chen, Junrong Wu, Liuyi Lu, Zuojian Hu, Xi Li, Li Huang, Xiaolian Zhang, Mingxing Chen, Xue Qin, Li Xie

**Affiliations:** ^1^Department of Clinical Laboratory, First Affiliated Hospital of Guangxi Medical University, Nanning, China; ^2^Department of Clinical Laboratory, Second Affiliated Hospital of Guangxi Medical University, Nanning, China; ^3^Department of Clinical Laboratory, Affiliated Tumor Hospital of Guangxi Medical University, Nanning, China

**Keywords:** hepatocellular carcinoma, immune infiltration, prognosis, tumor-associated macrophage, biomarker

## Abstract

**Aims:**

In the cancer-related research field, there is currently a major need for a greater number of valuable biomarkers to predict the prognosis of hepatocellular carcinoma (HCC). In this study, we aimed to screen hub genes related to immune cell infiltration and explore their prognostic value for HCC.

**Methods:**

We analyzed five datasets (GSE46408, GSE57957, GSE74656, GSE76427, and GSE87630) from the Gene Expression Omnibus database to screen the differentially expressed genes (DEGs). A protein–protein interaction network of the DEGs was constructed using the Search Tool for the Retrieval of Interacting Genes; then, the hub genes were identified. Functional enrichment of the genes was performed on the Metascape website. Next, the expression of these hub genes was validated in several databases, including Oncomine, Gene Expression Profiling Interactive Analysis 2 (GEPIA2), and Human Protein Atlas. We explored the correlations between the hub genes and infiltrated immune cells in the TIMER2.0 database. The survival curves were generated in GEPIA2, and the univariate and multivariate Cox regression analyses were performed using TIMER2.0.

**Results:**

The top ten hub genes [DNA topoisomerase II alpha (*TOP2A*), cyclin B2 (*CCNB2*), protein regulator of cytokinesis 1 (*PRC1*), Rac GTPase-activating protein 1 (*RACGAP1*), aurora kinase A (*AURKA*), cyclin-dependent kinase inhibitor 3 (*CDKN3*), nucleolar and spindle-associated protein 1 (*NUSAP1*), cell division cycle-associated 5 (*CDCA5*), abnormal spindle microtubule assembly (*ASPM*), and non-SMC condensin I complex subunit G (*NCAPG*)] were identified in subsequent analysis. These genes are most markedly enriched in cell division, suggesting their close association with tumorigenesis. Multi-database analyses validated that the hub genes were upregulated in HCC tissues. All hub genes positively correlated with several types of immune infiltration, including B cells, CD4^+^ T cells, macrophages, and dendritic cells. Furthermore, these hub genes served as independent prognostic factors, and the expression of these hub genes combing with the macrophage levels could help predict an unfavorable prognosis of HCC.

**Conclusion:**

In sum, these hub genes (*TOP2A*, *CCNB2*, *PRC1*, *RACGAP1*, *AURKA*, *CDKN3*, *NUSAP1*, *CDCA5*, *ASPM*, and *NCAPG*) may be pivotal markers for prognostic prediction as well as potentially work as targets for immune-based intervention strategies in HCC.

## Introduction

Hepatocellular carcinoma (HCC), the second leading cause of cancer-related death in the world, is a commonly fatal cancer with an unfavorable prognosis due to its complex genetics and clinical features (Siegel et al., 2015; Villanueva, 2019). To a certain extent, high heterogeneity contributes to the low survival rate of HCC despite excision, transplantation, transcatheter arterial chemoembolization, and radiofrequency ablation, among others, having been widely used in HCC treatment (Imamura et al., 2003; Forner et al., 2018; Caruso and Nault, 2019; Yin et al., 2019). Timely and effective intervention for HCC patients can improve not only their quality of life but also their survival rate (Deng et al., 2020; Kim et al., 2020). Therefore, the identification of new prognostic biomarkers and therapeutic targets plays a crucial role in HCC therapy.

Several prognostic biomarkers have been widely applied in HCC, such as alpha-fetoprotein and des-gamma-carboxyprothrombin (Fox et al., 2014; Abe et al., 2017). Nevertheless, these markers depend on the significant burthen of a tumor, which has resulted in their often limited application and inconsistent performance assessments (Park and Park, 2013). Numerous studies have demonstrated that valuable prognostic molecules can be identified from the bioinformatics analysis of high-throughput data, such as functional genes (Ma et al., 2020; Wang et al., 2020; Xie et al., 2020). From this, it can be determined that immune-associated genes may play a crucial role in HCC outcomes and targeted therapies on immune cells; thus, related genes have been developed for the reactivation of adaptive and innate immune systems and the creation of a strong antitumoral immune response. For instance, some researchers have found that inhibitors of programed death-1, programed death-ligand 1, and cytotoxic T lymphocyte-associated antigen 4 produce antitumoral effects on HCC cells (Langhans et al., 2019; Wang J. et al., 2019; Zhang Y. et al., 2020). Unfortunately, only sectional HCC patients with determinate tumor types react to the current immunotherapies; therefore, there is an urgent need to identify more underlying immune targets.

In the present study, differentially expressed genes (DEGs) were screened from the Gene Expression Omnibus (GEO) database. Then, we performed enrichment and protein–protein interaction (PPI) analyses of these genes to comprehend the functions of DEGs and identify the top ten hub genes in HCC. Next, we explored the potential correlations between each of the hub genes and infiltrated immune cells in the TIMER2.0 database. Furthermore, we visualized the prognostic landscape of candidate hub genes using several databases, including Oncomine (Rhodes et al., 2007), Gene Expression Profiling Interactive Analysis 2 (GEPIA2) (Tang et al., 2019), Human Protein Atlas (HPA) (Ponten et al., 2008), and TIMER2.0 (Li et al., 2020).

## Materials and Methods

### Data Source

The gene expression datasets (GSE46408, GSE57957, GSE74656, GSE76427, and GSE87630) of HCC were obtained from GEO^1^. All of the data included in the present study was available online. Information on these five datasets is summarized in Table 1.

**TABLE 1:** Details of GEO HCC data.

GEO	Platform	Tumor	Normal	Total number of samples	Number of identified DEGs
GSE46408	GPL4133	6	6	12	1,414
GSE57957	GPL10558	39	39	78	417
GSE74656	GPL16043	5	5	10	454
GSE76427	GPL10558	115	52	167	493
GSE87630	GPL6947	64	30	94	1,163

*GEO, Gene Expression Omnibus; HCC, hepatocellular carcinoma; DEGs, differentially expressed genes.*

### DEGs Processing

GEO2R^2^, an interactive online tool that can compare two different groups in a GEO dataset, was applied to screen the DEGs (Davis and Meltzer, 2007). The DEGs were defined as different expression genes between tumor and tumor-adjacent tissues in HCC patients with an adjusted *p* value < 0.05 and an absolute log fold-change (FC) > 1. Accordingly, to decrease the false discovery rate, the *p* values were adjusted using the Benjamini and Hochberg method. The overlapping up- and downregulated DEGs from these five datasets were identified using TBtools software (Chen et al., 2020).

### Gene Ontology (GO) and Kyoto Encyclopedia of Genes and Genomes (KEGG) Pathway Analyses

Functional enrichment analyses played a crucial role in the identification of biological characteristics in transcriptome data. In this study, the Metascape database was used to conduct the Gene Ontology (GO) terms and Kyoto Encyclopedia of Genes and Genomes (KEGG) pathway analyses in overlapping DEGs and identified hub genes (Zhou Y. et al., 2019). In order to choose the more remarkable terms within each cluster, a *p* value < 0.01 was considered statistically significant. We used bar charts and bubble diagrams, respectively, to visualize the results of the GO and KEGG analyses.

### PPI Network Construction and Module Analysis

The Search Tool for the Retrieval of Interacting Genes (STRING) is an interacting gene database designed to analyze PPI information (Szklarczyk et al., 2019). The overlapping DEGs were mapped in STRING to generate a network with functional interactions; then, this PPI network was visualized using Cytoscape software. Next, we employed the Molecular Complex Detection (MCODE) plugin to determine the most significant gene modules. Moreover, the cytoHubba plugin was applied to identify the hub genes using the Maximal Clique Centrality (MCC) method, and the top ten hub genes with the highest MCC scores were subjected to the subsequent analyses.

### Validation for mRNA and Protein Levels of Hub Genes in Multi-Databases

The Oncomine database^3^ is a publicly available cancer database that facilitates the analysis of genome-wide expression in multifarious cancers. In the present study, the Oncomine was utilized to overview the mRNA expression of candidate genes with a *p* value < 0.0001 and |FC| > 1.5.

The GEPIA2 database^4^, which includes TCGA and GTEx data, was applied to analyze the differential expression of the hub genes in the HCC and normal groups, and the cutoff values were set as |log2FC| = 1.0 and *p* value = 0.01.

Furthermore, immunohistochemistry analysis was conducted online to observe the distribution and protein level of the candidate hub genes in the HPA database^5^.

### Survival Analysis of Hub Genes

We employed GEPIA2 to perform overall survival (OS) and relapse-free survival (RFS) analyses, with a median group cutoff in 362 HCC patients. The survival curves with the calculated hazard rate (HR) and the log-rank *p* value were presented on the charts. Additionally, TIMER2.0 was utilized to conduct a univariate Cox analysis to validate the results from the GEPIA2 analyses.

### TIMER2.0 Database Analysis

TIMER2.0^6^ is a comprehensive source that can explore the relationship between two genes, and the correlations of mRNA expression and immune infiltration. In this study, we analyzed the correlations of hub gene expression and several tumor-associated immune cells, including B cells, CD8^+^ T cells, CD4^+^ T cells, macrophages, neutrophils, and dendritic cells (DCs). A *p* value less than 0.01 was considered statistically significant to identify the more prominent correlation between the hub genes and immune cells.

Further OS analyses were performed with macrophage and single hub gene expression. Moreover, we constructed ten multivariate Cox proportional hazard models, each of which comprised seven variables, including age, tumor stage, gender, race, tumor purity, macrophage level, and expression of the single candidate hub gene. The survival curves, featuring patterns of single gene expression, and macrophage levels were shown on the diagram. The association between each macrophage and OS was displayed as the low or high expression of a single hub gene.

### Statistical Analysis

The Student’s *t* test or non-parametric Mann–Whitney test was utilized to compare the two independent groups, as appropriate. The correlations between the candidate hub genes as well as the relationship of these genes and immune cell infiltration were assessed using Spearman’s correlation. The log-rank test was used to calculate the HR and log-rank *p* value to compare the survival curves. Univariate and multivariate Cox regression models were applied to calculate the HR and Cox *p* value. If not specifically stated, *p* values < 0.05 were considered statistically significant.

## Results

### Identification and Enrichment Analysis of DEGs

**FIGURE 1:**
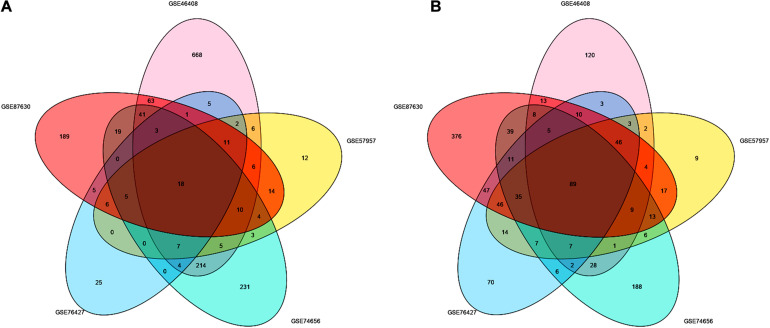
Identification of the DEGs between liver tumor and non-cancerous tissues in GSE46408, GSE57957, GSE74656, GSE76427, and GSE87630. Venn diagram of **(A)** upregulated and **(B)** downregulated DEGs based on the five GEO datasets. The overlapping areas represent the commonly altered DEGs. The *t*-test was used to analyze DEGs, with the cutoff criteria of |log FC| > 1.0 and adjusted *p* < 0.05. DEGs, differentially expressed genes; GEO, Gene Expression Omnibus; log FC, log fold change.

After screening the DEGs according to the criteria, 1,414, 417, 454, 493, and 1,163 genes were identified from GSE46408, GSE57957, GSE74656, GSE76427, and GSE87630, respectively (Table 1). These genes shared 107 DEGs among these five datasets, of which, there were 18 upregulated and 89 downregulated genes (Figures 1A,B and Supplementary Figure 1).

**FIGURE 2:**
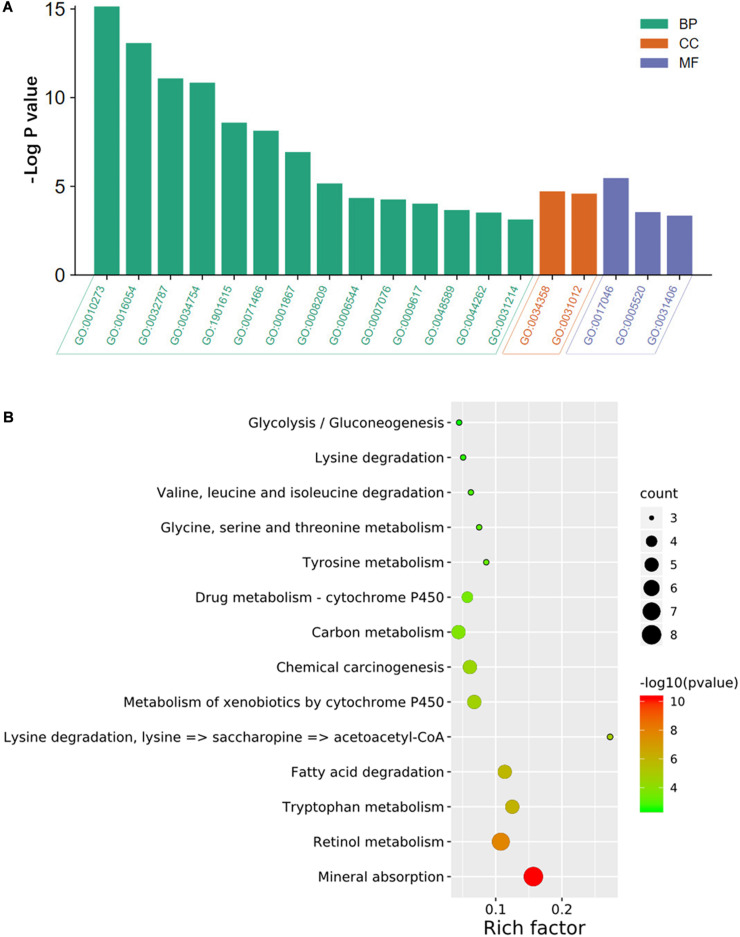
GO annotation and KEGG pathway enrichment analysis of DEGs. The significantly enriched **(A)** GO terms and **(B)** KEGG pathways. GO, gene ontology; KEGG, Kyoto Encyclopedia of Genes and Genomes; DEGs, differentially expressed genes; BP, biological process; CC, cellular component; MF, molecular function. GO:0010273, detoxification of copper ion; GO:0016054, organic acid catabolic process; GO:0032787, monocarboxylic acid metabolic; GO:0034754, cellular hormone metabolic process; GO:1901615, organic hydroxy compound metabolic process; GO:0071466, cellular response to xenobiotic stimulus; GO:0001867, complement activation, lectin pathway; GO:0008209, androgen metabolic process; GO:0006544, glycine metabolic process; GO:0007076, mitotic chromosome condensation; GO:0009617, response to bacterium; GO:0048589, developmental growth; GO:0044262, cellular carbohydrate metabolic process; GO:0031214, biomineral tissue development; GO:0034358, plasma lipoprotein particle; GO:0031012, extracellular matrix; GO:0017046, peptide hormone binding; GO:0005520, insulin-like growth factor binding; GO:0031406, carboxylic acid-binding.

As shown in Figure 2A, the results of the GO analysis suggested that the overlapping DEGs were principally enriched in the biological process (BP), especially in the detoxification of copper ion, the organic acid catabolic process, and the monocarboxylic acid metabolic process. In terms of cellular component (CC), the DEGs were enriched in plasma lipoprotein particles and extracellular matrix. In regard to molecular function (MF), the identified DEGs were significantly enriched in the aspects of peptide hormone binding, insulin-like growth factor binding, and carboxylic acid binding. Also, KEGG analysis showed that the overlapping DEGs were dramatically concentrated in mineral absorption and tryptophan metabolism (Figure 2B).

### PPI Network Construction and Hub Gene Identification

**FIGURE 3:**
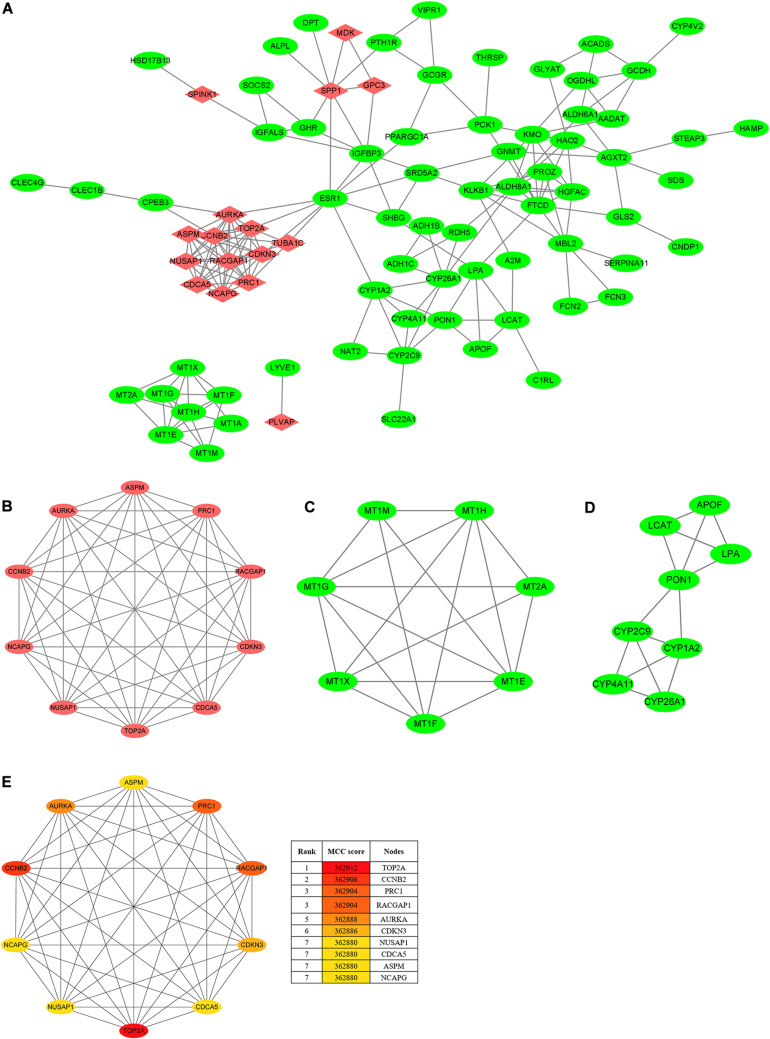
PPI network and clusters identification. **(A)** The PPI network of DEGs was constructed using Cytoscape. Upregulated genes are marked in red, and downregulated genes are marked in green. **(B)** Cluster 1 with 10 nodes and 45 edges. **(C)** Cluster 2 with 7 nodes and 18 edges. **(D)** Cluster 3 with 8 nodes and 14 edges. MCODE plugin was employed for the detection of clusters. Upregulated genes are marked in red, and downregulated genes are marked in green. **(E)** The top ten hub genes were identified using CytoHubba and ranked by the MCC score. The hub-gene network from CytoHubba analysis completely coincided with the Cluster 1 from MCODE analysis, both of which included the same 10 nodes and 45 edges. PPI, protein–protein interaction; DEGs, differentially expressed genes; MCODE, molecular complex detection; MCC, maximal clique centrality.

To seek the interactions of the overlapping DEGs, a PPI network, which included 84 nodes and 191 edges, was constructed and visualized in the Cytoscape (Figure 3A). As Figures 3B–D shows, the three most prominent subnetworks were identified using the MCODE plugin according to the connective degrees. Moreover, the CytoHubba plugin was used to determine the top ten hub genes based on their MCC scores (Figure 3E). Interestingly, the hub-gene network from the CytoHubba analysis completely coincided with the highest score module from the MCODE analysis, both of which included the same 10 nodes and 45 edges. Notably, DNA topoisomerase II alpha (*TOP2A*) was the most significant gene, with the highest MCC score of 362,912, followed by cyclin B2 (CCNB2) (MCC score = 362,906), protein regulator of cytokinesis 1 (*PRC1*) (MCC score = 362,904), Rac GTPase-activating protein 1 (*RACGAP1*) (MCC score = 362,904), aurora kinase A, (*AURKA*) (MCC score = 362,888), cyclin-dependent kinase inhibitor 3 (*CDKN3*) (MCC score = 362,886), nucleolar and spindle-associated protein 1 (NUSAP1) (MCC score = 362,880), cell division cycle-associated 5 (*CDCA5*) (MCC score = 362,880), abnormal spindle microtubule assembly (*ASPM*) (MCC score = 362,880), and non-SMC condensin I complex subunit G (*NCAPG*) (MCC score = 362,880). All the hub genes were upregulated in HCC tissues. In addition, the analyses of the correlations between candidate hub genes on the mRNA level were conducted on the TIMER2.0. Of these ten genes, every two genes showed a significant correlation (Spearman’s rho value > 0.7; *p* < 0.05) with or without tumor purity adjustment (Supplementary Table 1).

The high scores of these candidate hub genes indicated that there would be potential biological effects in the hub genes network; thus, we further determined the functional enrichment of these genes. The BP analysis proved that the hub genes were dramatically enriched in terms of cell division, the positive regulation of mitotic nuclear division, female gamete generation, and mitotic cell cycle phase transition. Besides, these genes were significantly enriched in terms of the mitotic spindle in the CC analysis and in terms of protein kinase binding in the MP analysis (Supplementary Figure 2). These results implied that these hub genes are closely associated with tumorigenesis.

### The Expression of Hub Genes Was Upregulated in Multi-Databases

**FIGURE 4:**
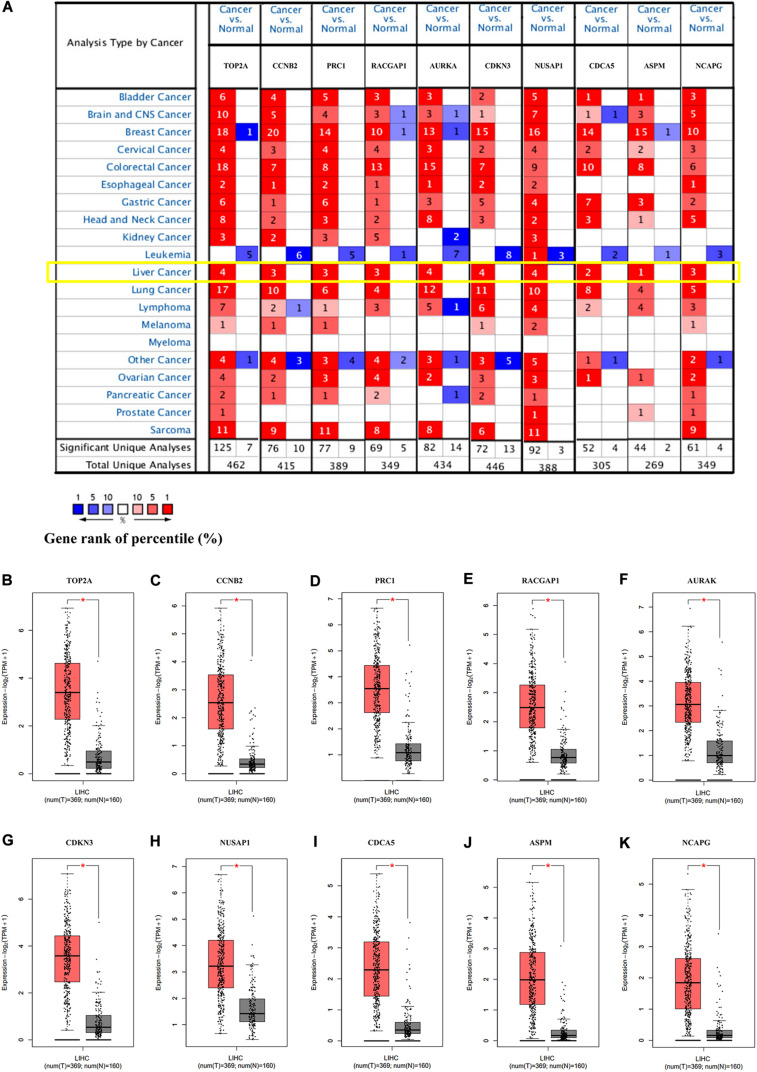
The expression of the hub genes was validated in HCC tissues. **(A)** The mRNA expression patterns of hub genes in the Oncomine database. This figure shows the numbers of datasets with statistically significant mRNA overexpression (red) or under-expression (blue) of the target gene (cancer vs. normal). The *p* value threshold is 0.0001. The number in each cell represents the number of analyses that met the threshold within the analyses and cancer types. The mRNA expression levels of **(B)** TOP2A, **(C)** CCNB2, **(D)** PRC1, **(E)** RACGAP1, **(F)** AURKA, **(G)** CDKN3, **(H)** NUSAP1, **(I)** CDCA5, **(J)** ASPM, and **(K)** NCAPG in LIHC tissues and normal liver tissues using GEPIA2. *p < 0.01 was considered statistically significant. HCC, hepatocellular carcinoma; LIHC, liver hepatocellular carcinoma. The “Yellow Box” specified the mRNA expression of these hub genes in liver cancer.

To verify the dependability of the results from the bioinformatics analysis, we next determined the mRNA expression levels of the hub genes in the Oncomine and GEPIA2 databases. The results showed that the transcriptional levels of *TOP2A*, *CCNB2*, *PRC1*, *RACGAP1*, *AURKA*, *CDKN3*, *NUSAP1*, *CDCA5*, *ASPM*, and *NCAPG* were significantly overexpressed in HCC tissue when compared with the normal controls (Figures 4A–K), indicating their potential oncogenic effects.

**FIGURE 5:**
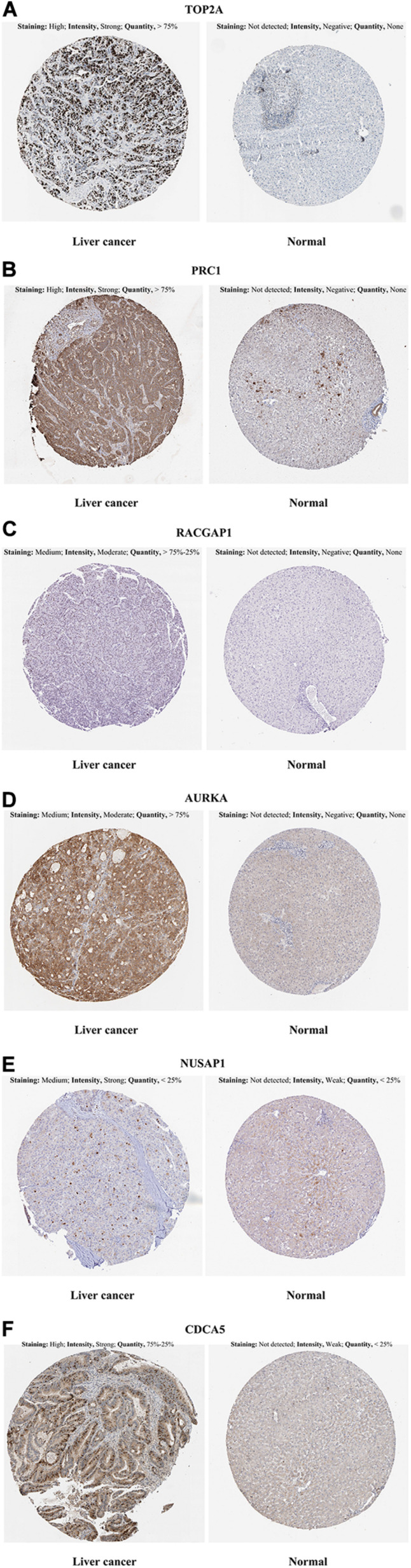
Representative immunohistochemistry images in HCC and normal liver tissues derived from the HPA database. **(A)** TOP2A incubating with HPA006458 antibody, **(B)** PRC1 incubating with HPA034521 antibody, **(C)** RACGAP1 incubating with HPA043912 antibody, **(D)** AURKA incubating with HPA002636 antibody, **(E)** NUSAP1 incubating with HPA042904 antibody, **(F)** CDCA5 incubating with HPA023691 antibody. HCC, hepatocellular carcinoma; HPA, Human Protein Atlas.

In the HPA database analysis, we found that the protein levels of TOP2A, PRC1, RACGAP1, AURKA, NUSAP1, and CDCA5 were significantly higher in the HCC tissues when compared to normal liver tissues (Figures 5A–F).

### The mRNA Levels of Hub Genes Are Positively Associated With Immune Infiltration in HCC

Numerous studies have demonstrated that the infiltration of tumor-associated immune cells and immune-related genes is correlated with the development and prognosis of HCC (Duan et al., 2019; Huang et al., 2020; Tang et al., 2020). Remarkably, targeting on these immune cells and/or genes has been a prospective workable approach in HCC therapy (Jayant et al., 2020). In this study, we attempted to explore the relationship between the mRNA expression of hub genes and immune cell infiltration using TIMER2.0. As Table 2 presents, each of the hub genes correlated with tumor purity in HCC tissues. Notably, we observed that these ten genes presented significant associations with infiltrating levels of B cells, CD4^+^ T cells, macrophages, and DCs, out of which these genes most strongly correlated with B cells [correlation coefficient (COR), 0.363 to 0.451; *p* < 0.01], macrophages (COR 0.262 to 0.384; *p* < 0.01), and DCs (COR, 0.449 to 0.548; *p* < 0.01), indicating that these hub genes were positively related to tumor-associated B cells, macrophages, and DCs in the HCC microenvironment.

**TABLE 2:** Correlation analysis between candidate hub genes and immune cells in the TIMER2.0 database.

Hub genes	Purity		B cell		CD8^+^ T cell		CD4^+^ T cell		Macrophage		Neutrophil		DC	
	COR	*P*	COR	*P*	COR	*P*	COR	*P*	COR	*P*	COR	*P*	COR	*P*
TOP2A	0.186	**	0.410	***	0.150	*	0.247	***	0.372	***	0.239	***	0.531	***
CCNB2	0.151	*	0.433	***	0.124	0.021	0.239	***	0.321	***	0.164	*	0.547	***
PRC1	0.191	**	0.426	***	0.142	*	0.247	***	0.384	***	0.217	***	0.526	***
RACGAP1	0.179	**	0.382	***	0.141	*	0.212	***	0.362	***	0.288	***	0.515	***
AURKA	0.148	*	0.451	***	0.058	0.280	0.157	*	0.262	***	0.096	0.076	0.466	***
CDKN3	0.183	**	0.403	***	0.123	0.022	0.188	**	0.311	***	0.128	0.018	0.449	***
NUSAP1	0.170	*	0.459	***	0.147	*	0.260	***	0.382	***	0.183	**	0.545	***
CDCA5	0.177	**	0.435	***	0.093	0.085	0.250	***	0.339	***	0.131	0.015	0.548	***
ASPM	0.169	*	0.364	***	0.148	*	0.210	***	0.294	***	0.203	**	0.453	***
NCAPG	0.146	*	0.407	***	0.095	0.078	0.203	**	0.314	***	0.177	**	0.509	***

COR, correlation coefficient; DC, dendritic cell; **, p < 0.0001; **, p < 0.001; *, p < 0.01.

### The Overexpression of Hub Genes Predicts Poor Prognosis in HCC

**FIGURE 6:**
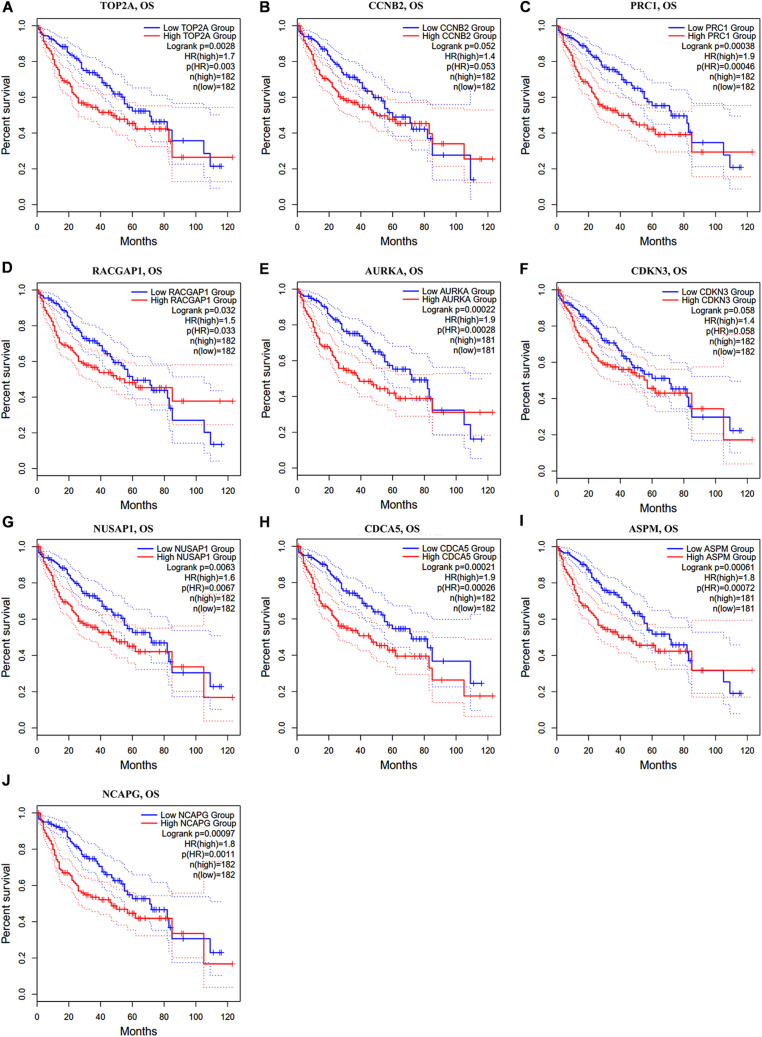
Overall survival analysis of ten hub genes in HCC patients. **(A)** TOP2A, **(B)** CCNB2, **(C)** PRC1, **(D)** RACGAP1, **(E)** AURKA, **(F)** CDKN3, **(G)** NUSAP1, **(H)** CDCA5, **(I)** ASPM, and **(J)** NCAPG. Log-rank *p* value < 0.05 was considered statistically significant. HCC, hepatocellular carcinoma; OS, overall survival; HR, hazard ratio.

**FIGURE 7:**
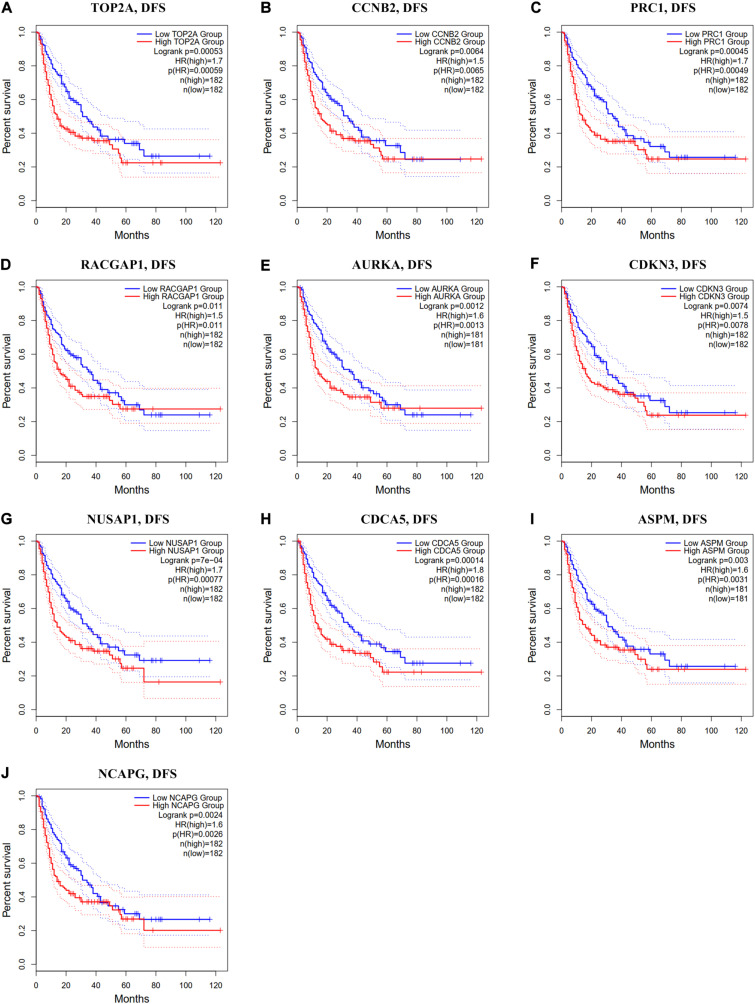
Disease-free survival analysis of ten hub genes in HCC patients. **(A)** TOP2A, **(B)** CCNB2, **(C)** PRC1, **(D)** RACGAP1, **(E)** AURKA, **(F)** CDKN3, **(G)** NUSAP1, **(H)** CDCA5, **(I)** ASPM, and **(J)** NCAPG. Log-rank *p* value < 0.05 was considered statistically significant. HCC, hepatocellular carcinoma; DFS, disease-free survival; HR, hazard ratio.

In the GEPIA2 analysis, the KM plotter analyses showed that the upregulated mRNA levels of *TOP2A*, *PRC1*, *RACGAP1*, *AURKA*, *NUSAP1*, *CDCA5*, *ASPM*, and *NCAPG* were correlated with worse OS in HCC patients; however, there was no significant correlation between *CCNB2/CDKN3* and HCC prognosis (Figures 6A–J). For the RFS analyses, the overexpression of all ten hub genes could predict unfavorable prognosis in HCC (Figures 7A–J). Additionally, we performed a univariate Cox regression analysis of the candidate hub genes in TIMER2.0. Table 3 summarizes the validation of the prognostic values of these hub genes in the TIMER2.0 database, suggesting that each of the hub genes may be an independent risk factor in HCC.

**TABLE 3:** Univariate Cox proportional hazards analyses of overall survival in the TIMER2.0.

Variables	HR	HR.95L	HR.95H	*p* value
TOP2A	1.245	1.112	1.394	0.000
CCNB2	1.274	1.119	1.451	0.000
PRC1	1.27	1.111	1.450	0.000
RACGAP1	1.417	1.206	1.664	0.000
AURKA	1.273	1.100	1.473	0.001
CDKN3	1.24	1.096	1.401	0.001
NUSAP1	1.208	1.056	1.381	0.006
CDCA5	1.397	1.202	1.625	0.000
ASPM	1.316	1.138	1.521	0.000
NCAPG	1.511	1.282	1.781	0.000

*HR, hazard rate.*

### The Overexpression of Hub Genes Accompanied by a High Level of Infiltrated Macrophages Predicts Poor Prognosis in HCC

**FIGURE 8:**
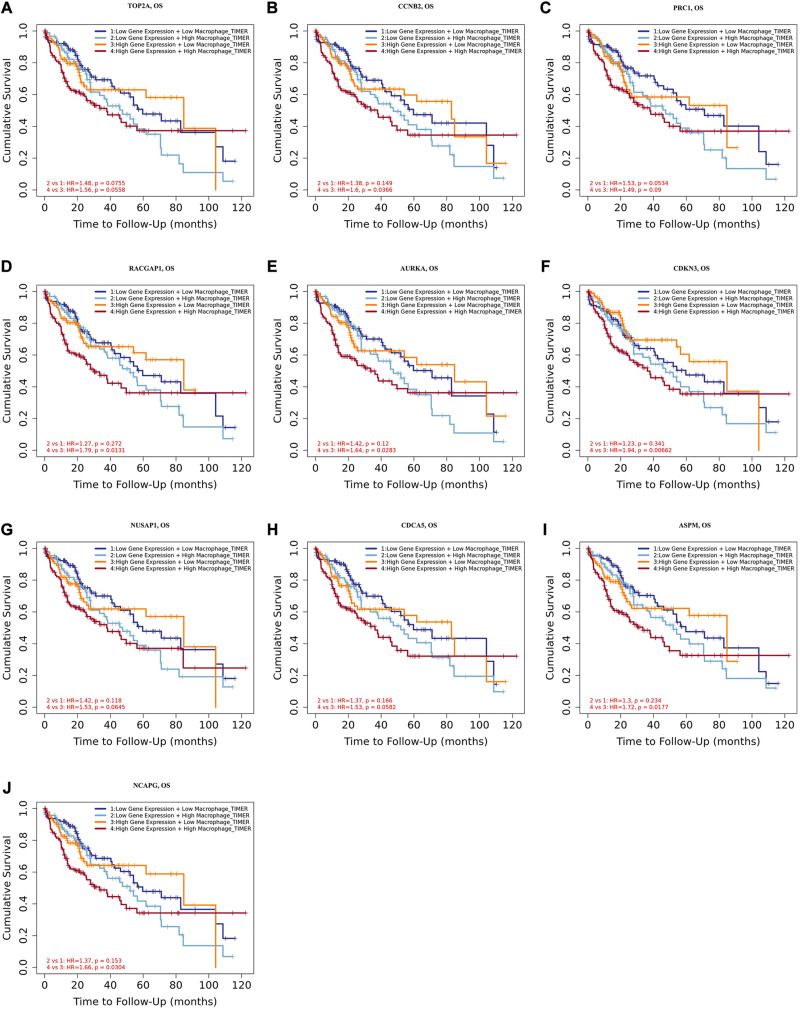
Overall survival analysis for combining the expression of single hub gene and macrophage in HCC patients. **(A)** TOP2A, **(B)** CCNB2, **(C)** PRC1, **(D)** RACGAP1, **(E)** AURKA, **(F)** CDKN3, **(G)** NUSAP1, **(H)** CDCA5, **(I)** ASPM, and **(J)** NCAPG. *P* value < 0.05 was considered statistically significant. HCC, hepatocellular carcinoma; HR, hazard ratio.

Tumor-associated macrophages (TAMs) majorly facilitate tumor angiogenesis, invasion, and metastasis and lead to a poor prognosis in HCC (Zhang et al., 2019; Zhao et al., 2020). There have been studies substantiating that targeting TAMs becomes a potential individualized precision single or combined therapy (Li et al., 2019). Therefore, the identification of TAM-related genes will contribute to providing more potential targets of the individualized precision treatment and improve the prognosis of HCC. In the present study, we evaluated the prognostic efficiency of the combination of TAMs and expression patterns for the single hub gene. As shown in Figures 8A–J, there was no significant relationship between the TAMs and prognosis under the low expression level of *TOP2A/CCNB2/PRC1/RACGAP1/AURKA/CDKN3/NUSAP1/CD CA5/ASPM/NCAPG*. However, under high *CCNB2* expression, higher macrophage levels had a worse outcome in HCC [HR = 1.6, *p* = 0.0366]. Similarly, the high macrophage level predicted unfavorable prognosis under the high expression of *RACGAP1* (HR = 1.79, *p* = 0.0131), *AURKA* (HR = 1.64, *p* = 0.0283), *CDKN3* (HR = 1.94, *p* = 0.0066), *ASPM* (HR = 1.72, *p* = 0.0177), and *NCAPG* (HR = 1.66, *p* = 0.0304).

**FIGURE 9:**
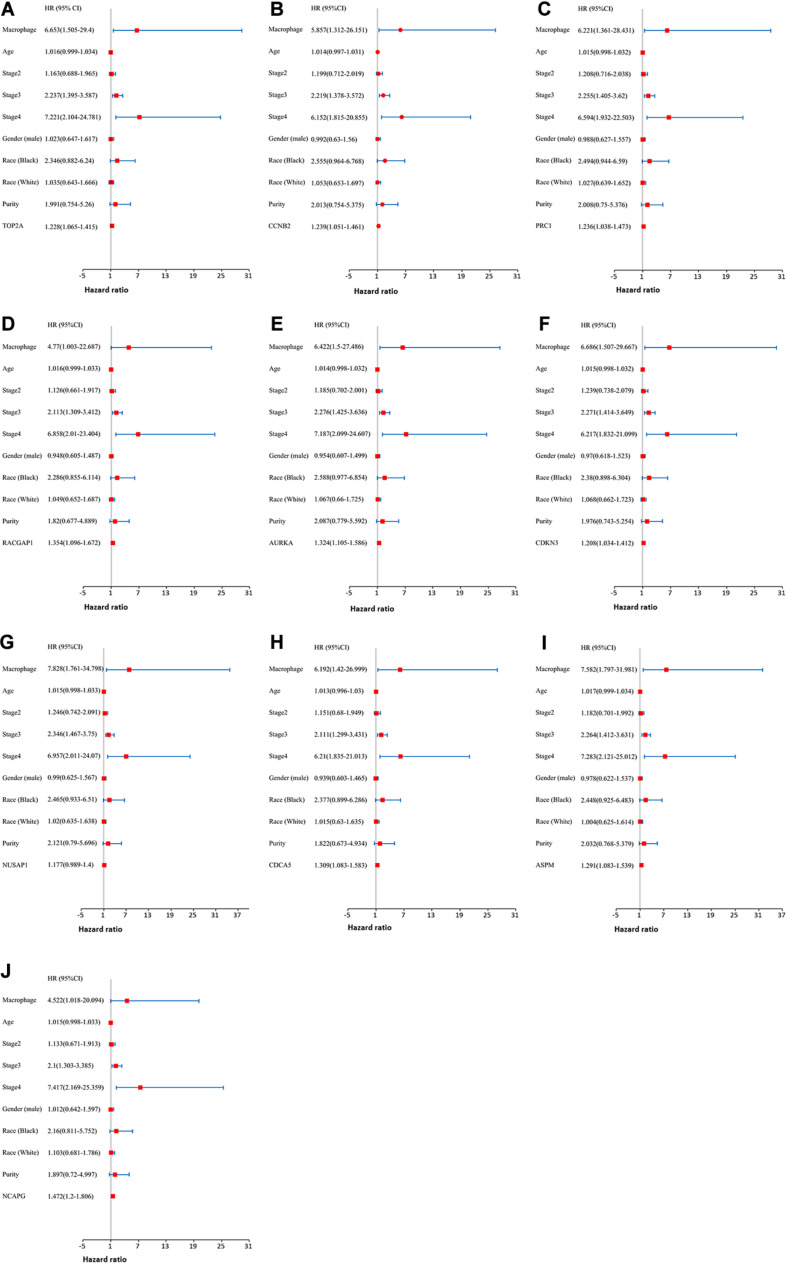
The univariate and multivariate Cox regression analysis of these hub genes. **(A)** TOP2A, **(B)** CCNB2, **(C)** PRC1, **(D)** RACGAP1, **(E)** AURKA, **(F)** CDKN3, **(G)** NUSAP1, **(H)** CDCA5, **(I)** ASPM, and **(J)** NCAPG. *P* value < 0.05 was considered statistically significant.

**FIGURE 10:**
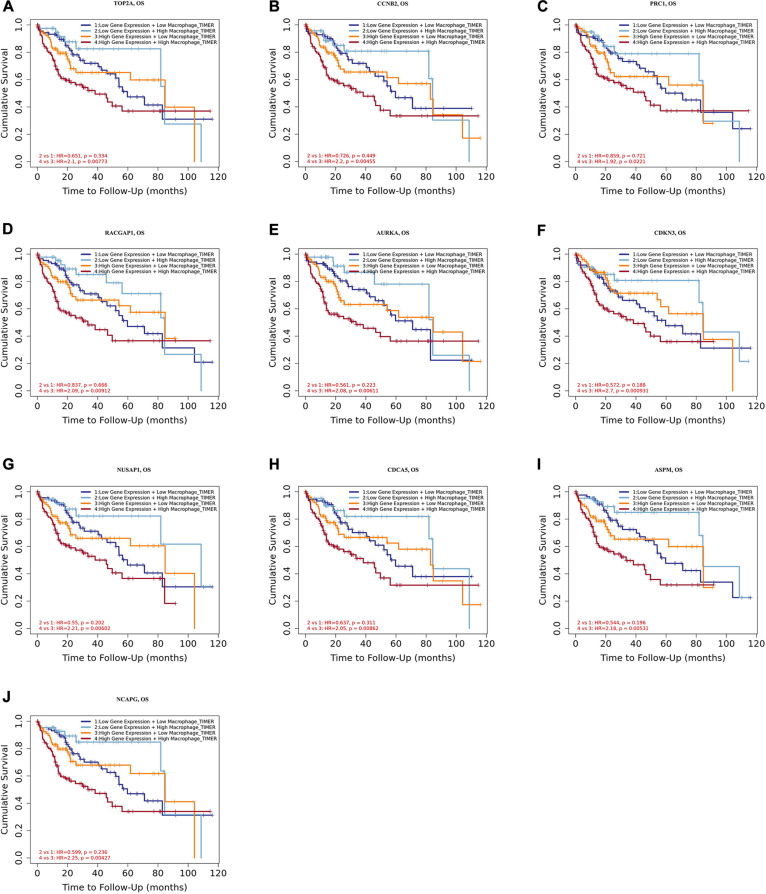
Overall survival analysis for combining the expression of single hub gene and macrophage in HCC patients after adjusting five confounding factors, including age, stage, gender, race, and tumor purity. **(A)** TOP2A, **(B)** CCNB2, **(C)** PRC1, **(D)** RACGAP1, **(E)** AURKA, **(F)** CDKN3, **(G)** NUSAP1, **(H)** CDCA5, **(I)** ASPM, and **(J)** NCAPG. Log-rank *p* value < 0.05 was considered statistically significant. HCC, hepatocellular carcinoma; HR, hazard ratio.

Moreover, we further established ten multivariate Cox regression analyses, each of which included seven variables: macrophage level, age, stage, gender, race, tumor purity, and expression of a single candidate gene (Figures 9A–J). The results showed that, after adjustments of age, stage, gender, race, and tumor purity, there was still no statistical correlation between TAM and prognosis with the low expression of *TOP2A/CCNB2/PRC1/RACGAP1/AURKA/CDKN3/NUSAP1/CD CA5/ASPM/NCAPG*; nevertheless, the lower level of TAM could predict favorable prognosis under the high expression of *TOP2A* (HR = 2.1, *p* = 0.0078)/*CCNB2* (HR = 2.2, *p* = 0.0046)/*PRC1* (HR = 1.92, *p* = 0.0221)/*RACGAP1* (HR = 2.09, *p* = 0.0091)/*AURKA* (HR = 2.08, *p* = 0.0061)/ *CDKN3* (HR = 2.7, *p* = 0.0009)/*NUSAP1* (HR = 2.25, *p* = 0.0043)/*CDCA5* (HR = 2.05, *p* = 0.0086)/*ASPM* (HR = 2.18, *p* = 0.0053)/*NCAPG* (HR = 2.25, *p* = 0.0043) (Figures 10A–J). These results suggested that each of the hub genes was an independent unfavorable prognostic biomarker and that combining their respective expression levels with the TAMs would help them play a more effective role in the prognosis prediction of HCC.

## Discussion

Hepatocellular carcinoma, one of the malignant cancers with high heterogeneity, is frequently diagnosed in its middle and advanced stages (Friemel et al., 2015; Buczak et al., 2018). Surgical resection remains the most crucial technique for HCC treatment; however, its therapeutic effects are always unsatisfactory (Abe et al., 2017; Deng et al., 2020). Thus, there is a need to screen novel carcinoma biomarkers and therapeutic targets. In the present study, comprehensive and bioinformatics analyses of multi-databases were applied to determine ten hub genes that appeared to be correlated with infiltrated immune cells in HCC. These genes were identified as independent prognostic factors in HCC patients.

In our study, five GEO datasets (GSE46408, GSE57957, GSE74656, GSE76427, and GSE87630) shared 107 common DEGs in HCC tissues; following this, the PPI network was constructed based on these genes. The results revealed a most significant module using the MCODE analysis that completely coincided with the subnetwork identification from the CytoHubba analysis. Particularly, ten hub genes in the module were upregulated in HCC tissues: *TOP2A*, *CCNB2*, *PRC1*, *RACGAP1*, *AURKA*, *CDKN3*, *NUSAP1*, *CDCA5*, *ASPM*, and *NCAPG*, respectively. The enrichment analyses presented that these hub genes were dramatically enriched in several terms of the BP analysis, including cell division, the positive regulation of mitotic nuclear division, female gamete generation, and the mitotic cell cycle phase transition. These genes were also significantly enriched in terms of the mitotic spindle in the CC analysis and in terms of the protein kinase binding in the MP analysis, suggesting that there is a close association between the hub genes and tumorigenesis. The validation in Oncomine and GEPIA2 confirms that the mRNA levels of *TOP2A*, *CCNB2*, *PRC1*, *RACGAP1*, *AURKA*, *CDKN3*, *NUSAP1*, *CDCA5*, *ASPM*, and *NCAPG* were significantly overexpressed in the HCC tissues, and at this validation, the *p* < 0.0001 and *p* < 0.01 were set in Oncomine and GEPIA2, respectively to more accurately identify the expression pattern of hub genes between HCC and normal tissues. Further HPA analysis also demonstrated that, compared to normal liver tissues, HCC tissues had significantly higher protein levels of TOP2A, PRC1, RACGAP1, AURKA, NUSAP1, and CDCA5, while we could not obtain the protein expression of CCNB2, CDKN3, ASPM, and NCAPG of HCC from the HPA website. These hub genes were validated to be closely correlated with infiltrated immune cells using the TIMER2.0 database. Both survival curves and univariate Cox regression analyses suggested that these candidate hub genes have a strong predictive ability for HCC. Previous studies demonstrated that TAMs extremely facilitate tumor angiogenesis and lead to a detrimental prognosis in HCC (Zhang et al., 2019; Zhao et al., 2020). The identification of TAM-related genes will facilitate providing more potential targets of the individualized precision treatment and improve the prognosis of HCC. Thus, we explored the prognostic value for the combination of candidate gene and TAM expression patterns in HCC and found that there was no significant relationship between TAMs and prognosis under a low expression level of the single hub gene. Meanwhile, under the high expression of *CCNB2/RACGAP1/AURKA/CDKN3/ASPM/NCAPG*, high TAM levels predicted unfavorable prognosis. Furthermore, the multivariate Cox regression models indicated that all candidate hub genes were independent predictors and that combining their respective expression levels with TAM will help them play a more effective role in the prognosis prediction of HCC.

In the enrichment analysis of candidate hub genes, we observed that nine out of ten genes were significantly associated with cell division: *TOP2A*, *CCNB2*, *PRC1*, *RACGAP1*, *AURKA*, *NUSAP1*, *CDCA5*, *ASPM*, and *NCAPG*. The remaining gene, *CDKN3*, has a dual function in the regulation of the cell cycle. On one hand, *CDKN3* serves as a cyclin-dependent kinase inhibitor while interacting with and dephosphorylating CDK2 kinase, thereby restraining its activation (Hannon et al., 1994; Johnson et al., 2002); on the other hand, CDKN3 can act as a MDM2-binding protein that forms a complex with MDM2 and P53, thus suppressing the production of P21, leading to the acceleration of cell cycle progression (Okamoto et al., 2006). These candidate hub genes have been demonstrated to work as oncogenes and are associated with clinical prognosis in numerous solid neoplasms, particularly in HCC (Roy et al., 2018; Gong et al., 2019; Lin J. et al., 2019). The *TOP2A* gene encoded a DNA topoisomerase, which controls and changes the topological status of DNA in the process of transcription and functions as a target for some antitumor agents (Delgado et al., 2018; Kitdumrongthum et al., 2020). Other bioinformatic analyses showed that *TOP2A* was related to development in cancers of the liver, esophagus, stomach, cervix, and lung, among others (Zeng et al., 2019; Kou et al., 2020; Zhang T. et al., 2020; Zhou et al., 2020). *CCNB2* is an important element for the process of cell cycle regulation. U2AF homology motif kinase 1 facilitates the nuclear enrichment of MYB proto-oncogene like 2 by affecting the expression of *CCNB2* to regulate cell cycle and proliferation (Wei et al., 2019), and reduced transmembrane protein 9 can contribute to decreased *CCNB2* levels and then promote cell cycle arrest in HCC cells (Zhang et al., 2016). *PRC1*, *RACGAP1*, and *CDCA5* were identified as the crucial genes in the pathological progression from cirrhosis to HCC, and their hypomethylation may drive the high expression of these genes (Lin Y. et al., 2019). *AURKA* can induce the metastasis of irradiated residuary HCC while promoting an epithelial-mesenchymal transition and cancer stem cell properties (Chen et al., 2017). *MYC* proto-oncogene and *AURKA* regulate the expression of each other at a mRNA level identified as a *MYC-AURKA* feedback loop (Lu et al., 2015). *CDKN3* overexpression can shorten the survival of HCC cells and shift sensitivity to chemotherapeutic drugs across the *AKT/P53/P21* signaling pathway; besides, *CDKN3* has been shown to be downregulated in advanced tumor stages (Dai et al., 2016). On the contrary, Chunyang et al. presented that the upregulation of *CDKN3* might facilitate cell proliferation via the stimulation of the G1-S transition (Xing et al., 2012). *NUSAP1* is a target for mir193A-5p, and evidence has shown that mir193A-5p might block the tumorigenesis of HCC through reducing *NUSAP1* (Roy et al., 2018). *ASPM* is associated with the development of HCV-related cirrhosis via the regulation of tumor-associated phosphorylation (Wang et al., 2017). *ASPM* is also considered a prognostic biomarker that predicts the increased possibility of invasive or metastatic HCC (Lin et al., 2008). *NCAPG* down-regulation indicates the suppression of HCC progression, possibly via the *PI3K-AKT* signaling pathway (Gong et al., 2019; Wang Y. et al., 2019). However, among these candidate hub genes, only *PRC1* has been reported to be an immune-related gene in a weighted gene co-expression network analysis (Wang et al., 2020).

Previous bioinformatics analyses have revealed that some *TOP2A*, *CCNB2*, *PRC1*, *RACGAP1*, *AURKA*, *NUSAP1*, *CDCA5*, *ASPM*, and *NCAPG* can be identified as key genes basing on different screening rules (Cai et al., 2019; Wang M. et al., 2019; Zhou Z. et al., 2019; Song et al., 2020). Comparing these previous studies, ours has the following advantages: First, this study included five GEO datasets, while others included two or three gene expression microarrays. In general, a greater number of included samples indicate more credible results in integrated research. Second, we constructed ten gene-macrophage Cox regression models. Inevitably, there were still several limitations of the present study. The included datasets came from different platforms, which might lead to an uncertain systematic bias. Furthermore, TIMER2.0 is a visual website based on tumor tissue information from the Cancer Genome Atlas database (Li et al., 2020). Thus, although tumor purity adjustment was performed in the correlation analyses between the immune cell and candidate genes, there was still systematic bias. To overcome this issue, the application of single-cell RNA sequencing at a higher resolution should be conducted (Papalexi and Satija, 2018). Finally, future experiments *in vivo*/*in vitro* should be performed to verify the results of this bioinformatics analysis.

Numerous studies have revealed that immune cell infiltration, TAMs, for instance, can serve as a biomarker for the diagnosis and prognosis of various cancers (Ali et al., 2016; Zhou R. et al., 2019). Thus, we assessed the prognostic value of the combination of TAMs and expression patterns for each of the hub genes. The results showed that the high TAM level predicted unfavorable prognosis under the condition of the high expression of the hub genes, while there was no significant correlation between the TAM and prognosis under the condition of the low expression level of these genes. These results suggested that the combination of hub-gene expression and the TAM levels would play a more effective role in the prognosis prediction of HCC.

In summary, we identified ten genes with a positive correlation with infiltrated-immune cells. These candidate genes present the marked prognostic value of HCC and act as independent prognosis factors for patients with HCC. Moreover, these genes may function in the progression of HCC. Furthermore, we determined that combining the expression of these genes and TAMs can provide a more efficient HCC prognosis prediction. Overall, these findings suggest that these hub genes may be potential targets of immune therapy.

## Data Availability Statement

The datasets presented in this study can be found in online repositories. The names of the repository/repositories and accession number(s) can be found in the article/Supplementary Material.

## Author Contributions

XQ and LX: conceptualization. XL and LH: GEO searches and analyses. JW, ZH, and MC: transcriptome data visualization and validation. HC: writing. HC, JW, and LL: proofreading. All authors have read and approved the manuscript.

## Conflict of Interest

The authors declare that the research was conducted in the absence of any commercial or financial relationships that could be construed as a potential conflict of interest.

## Footnotes

1 http://www.ncbi.nlm.nih.gov/geo

2 https://www.ncbi.nlm.nih.gov/geo/geo2r/

3 https://www.oncomine.org/

4 http://gepia2.cancer-pku.cn

5 https://www.proteinatlas.org/

6 http://timer.cistrome.org/

## Abbreviations

HCC: hepatocellular carcinoma
DEGs: differentially expressed genes
GEO: Gene Expression Omnibus
PPI: protein–protein interaction
GEPIA2: Gene Expression Profiling Interactive Analysis 2
HPA: human protein atlas
GO: Gene Ontology
KEGG: Kyoto Encyclopedia of Genes and Genomes
STRING: Search Tool for the Retrieval of Interacting Genes
BP: biological process
CC: cellular component
MF: molecular function
TOP2A: DNA topoisomerase II alpha
CCNB2: cyclin B2
PRC1: protein regulator of cytokinesis 1
RACGAP1: Rac GTPase-activating protein 1
AURKA: aurora kinase A
CDKN3: cyclin-dependent kinase inhibitor 3
NUSAP1: nucleolar and spindle-associated protein 1
CDCA5: cell division cycle-associated 5
ASPM: abnormal spindle microtubule assembly
NCAPG: non-SMC condensin I complex subunit G
DC: dendritic cell
COR: correlation coefficient
OS: overall survival
RFS: relapse-free survival
TAM: tumor-associated macrophage
HR: hazard rate
FC: fold change
MCODE: Molecular Complex Detection
MCC: Maximal Clique Centrality.
