# Supplementary figures and images for: Identification of Hub Genes Associated With Immune Infiltration and Predict Prognosis in Hepatocellular Carcinoma via Bioinformatics Approaches
Source: Front Genet. 2021 Jan 11;11:575762. doi: 10.3389/fgene.2020.575762 (PMC7831279; doi:10.3389/fgene.2020.575762)

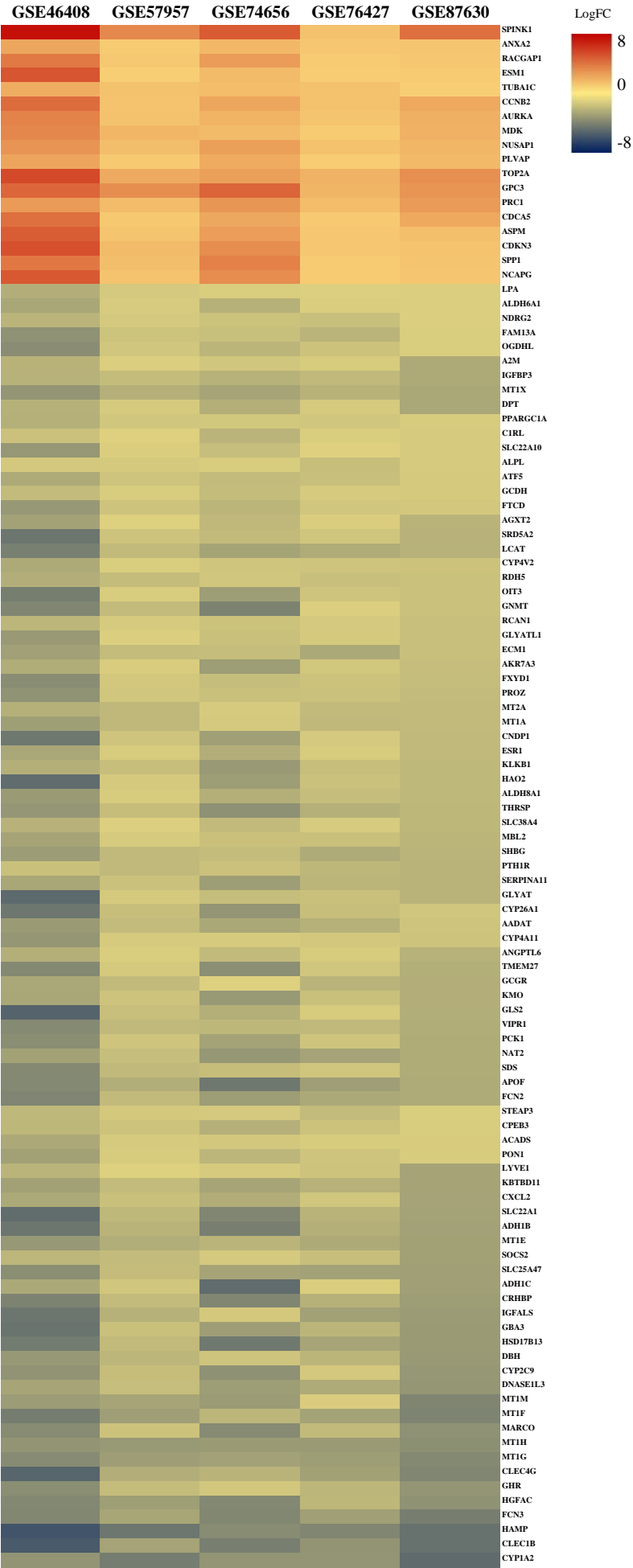

Supplement: Supplementary file 1 [file Image_1.pdf]

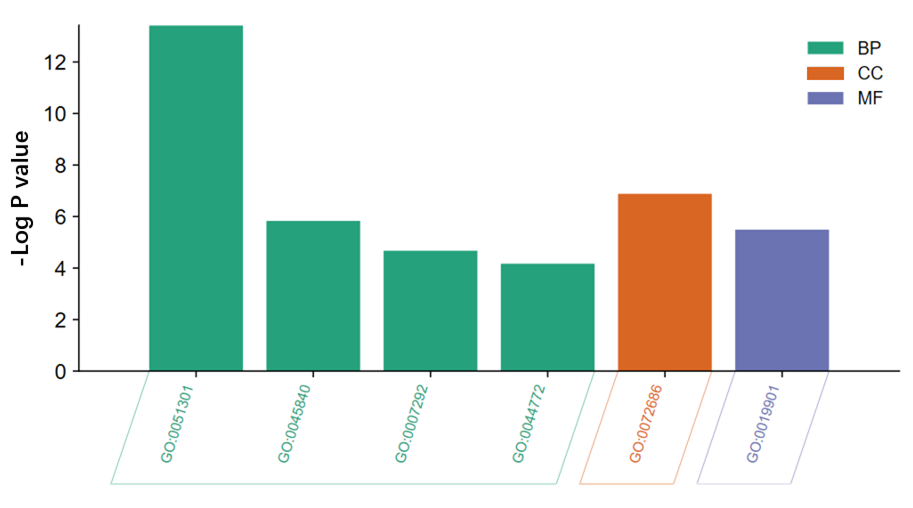

Supplement: Supplementary file 2 [file Image_2.tif]
