# Supplementary material for: Identification of Hub Genes Associated With Immune Infiltration and Predict Prognosis in Hepatocellular Carcinoma via Bioinformatics Approaches
Source: Front Genet. 2021 Jan 11;11:575762. doi: 10.3389/fgene.2020.575762 (PMC7831279; doi:10.3389/fgene.2020.575762)
Supplement: Supplementary file 4 [file Data_Sheet_1.docx]

**Supplementary Figure Legends**

**Supplementary Figure 1.** Heatmap of the overlapping DEGs. Blue represents a low log FC value, and red represents a high log FC value. Each column represents one dataset, and each row represents one gene. The gradual color change from red to blue represents the changing process from up-regulation to down-regulation. DEGs, differentially expressed genes; log FC, log‑fold change.

**Supplementary Figure 2.** GO enrichment analysis of ten hub genes. GO:0051301, cell division; GO:0045840, positive regulation of mitotic nuclear division; GO:0007292, female gamete generation; GO:0044772, mitotic cell cycle phase transition; GO:0072686, mitotic spindle; GO:0019901, protein kinase binding.
